# Winners of Lasker Foundation Video Contest announced

**DOI:** 10.1172/JCI210286

**Published:** 2026-07-15

**Authors:** Mitch Leslie

This year the Lasker Foundation held its first video contest to encourage graduate and medical students and early-career scientists to build skills for communicating important medical and scientific issues to broad audiences. Limited to no more than 2 minutes in length, the videos describe a biomedical research question the creator is studying (or would like to study) and how it could or should be addressed to benefit science and patients in the future. The contest drew 124 entries from 15 countries. Forty-one of the entrants were graduate students, another 31 were medical students, and 15 were postdoctoral fellows.

Today, the Lasker Foundation announced the top 6 videos. The winners’ institutions will receive $5,000 to cover their educational expenses. In addition, the winners will be invited to attend the Lasker Luncheon, at which the recipients of this year’s Lasker Awards are celebrated.

The Video Contest winners are Colette Benko, Max Bucklan, Lindsey Ramirez, Dimitri Seneviratne, Mark Sorin, and Sai Krishna Bhamidipati, and their winning videos can be found at the Lasker Foundation (https://laskerfoundation.org/meet-the-winners-of-the-2026-video-contest).

Born and raised in Calgary, Colette Benko (for video, see https://youtu.be/N7bkYvuxB3M) has been honing her science communication skills by leading tours of the elephant seal colony at Año Nuevo State Park in California. She says that her colleagues encouraged her to enter the contest after she used her own cartoons in a lab presentation. Benko, a fourth-year PhD student at Stanford University who is studying how cells signal their internal state to the immune system, took their advice. “It was a great opportunity to describe the work we are doing and share the molecular biology behind it.” Benko says she wants to continue spreading the word about research as she continues her career. “There are always places to share a love of science.”

Max Bucklan (see https://youtu.be/orbdyT5L5Xw), who grew up in Connecticut, is an experienced video creator and editor who minored in film and video as an undergraduate at Duke University. When he saw the announcement about the contest, “I said I have got to do this.” Bucklan, a third-year PhD student at Duke, is studying different RNA versions produced from the same gene to better understand how these variants can lead to diseases. To illustrate some of the ideas in his video, Bucklan enlisted Lego bricks, filming intricate stop-action animations that took hours to complete. “I love being creative,” he says.

For Chicago native Lindsey Ramirez (see https://youtu.be/cDhXzl4hBsU), the video contest was appealing because “the part of science I really love is to translate it and make it approachable for a lot of people.” She is currently a postdoctoral researcher at the University of Illinois College of Medicine, where she investigates the effects of alcohol on the brain and the mechanism that tells us when to stop drinking. Ramirez had created videos before, mainly to explain her lab work, but producing an accurate, clear, and compelling two-minute video was tough, she says. “The first video I did was five minutes, and it seemed insanely short.”

The contest was a natural choice for Wichita-born Dimitri Seneviratne (see https://youtu.be/GzcdAkFnO74). “If I get the chance to make videos, I’ll always do it,” he says. Both of his parents are engineers, and he intended to become one as well, majoring in product design and manufacturing engineering at Wichita State University. But a visit to Sri Lanka, his parents’ birthplace, in 2023 opened his eyes to the challenges that many people face in obtaining quality, trustworthy medical care. He decided to switch to medicine and is now in his first year of medical school at the University of Kansas School of Medicine.

Unlike most of his fellow winners, Mark Sorin (see https://youtu.be/KqzO8VqKWcU) was a video newbie when he heard about the contest. But the press had covered research from the lab where he earned his MD-PhD degree at McGill University, so he understood the importance of clearly conveying scientific findings. He decided to enter the contest because “I thought it was a very interesting challenge.” Sorin, who was born and raised in Montreal, is now a medical resident at McGill. He chose artificial intelligence to make the animations for his video because “I like to stay up to date and play around with and use new tools,” he says.

PhD student Sai Krishna Bhamidipati (see https://youtu.be/GAYP8uOF69U) of the University of California, San Diego, also had never made a video — or even considered making one. But Bhamidipati, who studies how inhibitory neurons work together to control brain activity, decided to make his first foray into video production because the contest would test his ability “to distill ideas in an accessible manner.” The key to making his research understandable, he decided, was to choose “a watertight metaphor.” His video likens the brain to a city with traffic signals that represent inhibitory or stimulatory neurons. Bhamidipati says he’d like to continue explaining science but not necessarily through videos. “The message is more important than the medium.”

## Version 1 07/15/2026

Electronic publication

